# Natural Compounds as Metabolic Modulators of the Tumor Microenvironment

**DOI:** 10.3390/molecules26123494

**Published:** 2021-06-08

**Authors:** Ana S. Dias, Luisa Helguero, Catarina R. Almeida, Iola F. Duarte

**Affiliations:** 1 Department of Chemistry, CICECO—Aveiro Institute of Materials, University of Aveiro, 3810-193 Aveiro, Portugal; a.dias@ua.pt; 2 Department of Medical Sciences, iBiMED—Institute of Biomedicine, University of Aveiro, 3810-193 Aveiro, Portugal; luisa.helguero@ua.pt (L.H.); cra@ua.pt (C.R.A.)

**Keywords:** tumor microenvironment, stromal cells, metabolism, metabolic modulation, natural compounds, phytochemicals, cancer

## Abstract

The tumor microenvironment (TME) is a heterogenous assemblage of malignant and non-malignant cells, including infiltrating immune cells and other stromal cells, together with extracellular matrix and a variety of soluble factors. This complex and dynamic milieu strongly affects tumor differentiation, progression, immune evasion, and response to therapy, thus being an important therapeutic target. The phenotypic and functional features of the various cell types present in the TME are largely dependent on their ability to adopt different metabolic programs. Hence, modulating the metabolism of the cells in the TME, and their metabolic crosstalk, has emerged as a promising strategy in the context of anticancer therapies. Natural compounds offer an attractive tool in this respect as their multiple biological activities can potentially be harnessed to ‘(re)-educate’ TME cells towards antitumoral roles. The present review discusses how natural compounds shape the metabolism of stromal cells in the TME and how this may impact tumor development and progression.

## 1. Introduction

The tumor microenvironment (TME) can be defined as the complex and dynamic milieu where cancer cells are embedded. It comprises nonmalignant cells, such as infiltrating immune cells, fibroblasts, endothelial cells, and adipocytes, together with the extracellular matrix and a variety of cytokines, chemokines, and growth factors resulting from heterotypic signaling. All these components actively interact and contribute to an evolving balance between anti- and protumoral events [1]. For instance, immune cells recruited to the tumor site (e.g., monocytes/macrophages and lymphocytes) can either help to eliminate cancer cells, mainly in early stages of tumor development, or perform protumorigenic functions via multiple mechanisms. Nonimmune stromal cells are also key for cancer cells to thrive, as evidenced by the role of activated fibroblasts in ECM remodeling to favor cell invasion and migration or the involvement of endothelial cells in tumor vascularization needed to supply oxygen and nutrients to cancer cells, clear metabolic waste, and enable tissue invasion by metastatic cells. Besides supporting tumor growth and progression, the TME strongly determines the success of anticancer therapies, mainly by physically influencing drug access and inducing drug resistance through soluble factors, cell–cell interactions, and/or immune responses [2]. Hence, due to its well-established importance in cancer progression and response to treatment, the TME is currently considered a central paradigm in oncobiology and anticancer drug development. 

Metabolic reprogramming is widely accepted as one of the major cancer hallmarks [3,4]. Tumor cells typically show altered uptake and metabolic processing of nutrients, mainly to sustain their enhanced energetic and biosynthetic needs, as well as to maintain a favorable balance between the production of reactive oxygen species (ROS) and antioxidant mechanisms [5]. Rewired metabolisms of tumor cells stem from changes in signaling pathways, protein expression, and other molecular mechanisms but is also strictly linked to the interplay with other cells in the TME via paracrine signaling, competition for nutrients, and cooperative metabolic exchange [6]. For instance, lactate produced by glycolytic cancer cells and activated fibroblasts may serve as metabolic fuel for less glycolytic tumor cells. Moreover, lactate-induced acidification favors metastasis, angiogenesis, immune evasion, and immunosuppression [7]. On the other hand, the metabolic programs adopted by stromal cells, in response to tumor signals and the changing microenvironment, strongly determine the phenotypic and functional features of TME cells, hence their contribution to tumor development and progression [8]. Consequently, modulating the metabolism of the TME cells has emerged as an attractive strategy to hinder the protumoral roles of these cells or even to ‘(re)-educate’ them towards antitumoral functions.

Several natural compounds produced by plants, microorganisms and marine organisms, which display strong cytotoxic activity against a variety of tumor cells, are under preclinical testing or used already as conventional chemotherapy drugs [9]. The enormous structural diversity, adequacy to chemical modification, and multitargeting activities of these compounds are some of the features that make them attractive as anticancer cytotoxic and/or cytostatic agents. Moreover, many of these molecules have great potential to sensitize cancer cells to different therapeutic approaches, including radiotherapy, chemotherapy, and immunotherapy, as recently reviewed for flavonoids [10]. Notably, besides direct effects in cancer cells, some natural compounds exhibit other biological activities in noncancerous cells, such as antioxidant, anti-inflammatory, and immunomodulatory activities that empower the host immune system, enhance the efficacy of anticancer drugs, and/or protect normal cells from drug toxicity [11,12]. Consequently, a growing number of studies is now focusing on the effects of natural compounds beyond tumor cells, with special emphasis on the modulation of immune cells in the TME [13,14]. Given the relevance of cell metabolism in the TME, the purpose of the present review is to specifically address how natural compounds shape the metabolism of key noncancer cells in the TME and how this may impact tumor development and progression. We will start by a brief description of TME cellular components and their metabolic plasticity, followed by illustrative examples of antitumoral metabolic reprogramming mediated by natural compounds. This knowledge, combined with our understanding on the direct effects of these compounds in the metabolism of tumor cells (recently reviewed in [15,16]), is expected to provide a more integrated picture of the biological impact and therapeutic potential of these compounds as anticancer metabolic modulators.

## 2. Cells in the Tumor Microenvironment and Their Metabolic Plasticity

### 2.1. Malignant Cells

Extensive research on metabolic reprogramming of tumor cells has identified key hallmarks of oncometabolism, which are commonly found across different cancer types and known to actively contribute to tumorigenesis. Their detailed description can be found in excellent recent reviews [17,18]. In general terms, the main metabolic features of cancer cells include the overutilization of glucose and upregulation of aerobic glycolysis, which leads to augmented lactate secretion (Warburg effect), along with increased glutaminolysis and the upregulation of pathways essential to the biosynthesis of macromolecules and/or to redox control (e.g., pentose phosphate pathway, one-carbon metabolism, and de novo lipid synthesis). Furthermore, it has come to light those niches of cancer cells in different parts of the tumor can have different metabotypes that sustain symbiotic relationships and favor tumor growth. For instance, the lactate secreted by highly glycolytic cancer cells, typically located in strongly hypoxic regions, can be used by more oxidative cancer cells, which convert it into pyruvate to fuel the tricarboxylic acid cycle (TCA cycle) and mitochondrial energy production [7]. 

The tumor metabolic heterogeneity is further augmented by the presence of small groups of stem-like cancer cells in the TME. These so-called cancer stem cells (CSCs) have the ability to self-renew and a high tumorigenic activity, thus being chiefly implicated in disease progression, metastasis to distant sites, and resistance to chemo-/radiation therapies [19,20]. In fact, the elimination of CSCs is currently viewed as a key strategy in cancer treatment and the prevention of metastatic disease. CSCs share several properties with normal stem cells, such as the expression of some surface markers (e.g., cluster of differentiation CD44 and CD133 or the enzyme aldehyde dehydrogenase isoform 1, ALDH1); activation of particular cell-signaling pathways (Wnt, Notch, or Hedgehog); quiescence for long periods of time; and active DNA repair capacity, which enable them to regenerate the tumor mass. It has recently come to light that the stemness and tumorigenic potential of CSCs are closely linked to their metabolism [21]. While there is no consensus on the dominant metabolic phenotype of CSCs, mounting evidence suggests that these cells have great metabolic plasticity in response to microenvironmental conditions and that different CSC subpopulations may display distinct metabolic programs. Compared to their differentiated progeny, CSCs appear to rely more on mitochondrial respiration and less on aerobic glycolysis for energy production, as sustained by increased mitochondrial mass and oxygen consumption [22,23]. A functional oxidative phosphorylation, together with a tight control of the cell’s redox status, have been identified as crucial to maintain CSCs stemness and metastatic potential. Indeed, these cells are highly vulnerable to drugs that inhibit mitochondrial respiration, such as metformin [24,25]. On the other hand, the ability to adapt to hypoxia, starvation, and/or the pharmacological inhibition of oxidative phosphorylation (OXPHOS), by boosting the glycolytic phenotype and adopting a mixed glycolytic/respiratory phenotype, enables CSC maintenance and therapy resistance [21].

### 2.2. Immune Cells

#### 2.2.1. Tumor-Associated Macrophages

Tumor-associated macrophages (TAM) are one of the most abundant immune cell populations infiltrating the TME [26]. Macrophages are highly plastic phagocytic cells that can polarize to a continuum spectrum of activation states, depending on the microenvironmental stimuli. Inflammatory M1-like macrophages display tumoricidal functions, but the anti-inflammatory M2-like macrophages, that can be induced by soluble factors present in the TME, are essentially protumorigenic [27,28]. As a positive correlation between M2-like TAM frequency and worse prognosis has been established in several cancer types [29,30,31,32], shifting TAM polarization towards an antitumoral M1-like phenotype has emerged as an attractive strategy to elicit tumor regression and aid cancer treatment. Importantly, macrophage phenotypes are linked to their metabolic programs. Proinflammatory M1-like macrophages are typically highly glycolytic with impairment of the TCA cycle, leading to accumulation of citrate and succinate. In contrast, the metabolism of M2-like macrophages mainly relies on the TCA cycle and OXPHOS. Evidence suggests that targeting macrophage energetic metabolism impacts on tumor growth. For example, hampering the function of immune-responsive gene 1 (Irg1), which encodes an enzyme synthesizing itaconate from the TCA cycle intermediate cis-aconitate, in tumor-resident macrophages, leads to reduced itaconate production and decreases tumor progression [33].

The production of nitric oxide (NO) via the upregulated inducible nitric oxide synthase (iNOS)-mediated catabolism of arginine is another crucial feature of macrophage metabolism, especially of M1-like macrophages. Conversely to M2-like macrophages, which display upregulated arginase 1 (ARG1), an enzyme that catalyzes the conversion of L-arginine to L-ornithine and urea, M1 cells can generate high amounts of NO upon metabolizing L-arginine [34,35]. This NO is associated with the microbicidal activity of M1 macrophages. In the TME, NO production may display either pro- or antitumoral activities, depending on the concentrations reached and the cell types involved [36]. Indeed, the role of NO in modulating TAM behavior and its interactions with other TEM cells is multifaceted. Broadly, high levels of NO are usually associated with cellular apoptosis, while low levels of NO promote tumorigenesis [37].

#### 2.2.2. T Lymphocytes

T cells play a significant role in antitumor immunity, mostly through their capacity to lyse cancer cells. There are several types of T lymphocytes, including cytotoxic T cells (Tc) and regulatory T cells (Treg), among others [38]. Tc have antitumoral activities, whereas Treg inhibit Tc activity to suppress immunity. Thus, high levels of Treg are correlated with a poor prognosis in many cancers [39]. The metabolic requirements of T cells change from naïve to effector T cells. These activated lymphocytes mainly rely on aerobic glycolysis to obtain energy, displaying increased glucose uptake and flux through the pentose phosphate pathway (PPP), as well as increased glutamine metabolism [40,41,42]. Tc function may be hampered by the depletion of amino acids and glucose from the TME by cancer cells. Conversely, it was found that reprogramming Tc activity by promoting the production of the glycolytic intermediate PEP (phosphoenolpyruvate) leads to more efficient Tc function, associated with restricted tumor growth [43]. On the other hand, anti-inflammatory Treg cells and CD8+ memory T cells rely on OXPHOS to survive [40].

#### 2.2.3. Natural Killer Cells

Natural Killer (NK) cells are another type of cytotoxic lymphocytes that play an important role in antitumor immune responses. Results from a recent meta-analysis have concluded that high levels of NK cell markers (CD56, CD57, NKp30, and NKp46) are significantly correlated with favorable prognosis in patients with solid tumors [44]. To enhance their cytotoxic function, activated NK cells upregulate glycolysis and OXPHOS [45]. Thus, the competition for glucose with cancer cells might inhibit NK cell activation. Additionally, lactate accumulation and lower pH in the TME also result in decreased NK cell activity [46,47].

#### 2.2.4. Dendritic Cells

Mature dendritic cells (DCs) are antigen-presenting cells (APCs) that activate naïve T lymphocytes and play a critical role in inducing adaptive immune responses. On the other hand, DCs can adopt a tolerogenic profile and inhibit the immune response. In tumors, there is typically an accumulation of tolerogenic DCs [48]. Expression of the enzyme indoleamine 2,3-dioxygenase (IDO) is particularly significant, because IDO activity converts mature DCs into tolerogenic APCs that promote Tregs and suppress Tc [49,50,51]. DC activation mainly relies on glycolysis during activation [52], whereas fatty acid oxidation drives DCs towards a tolerogenic phenotype by increasing the flux through the TCA cycle [53].

### 2.3. Nonimmune Stromal Cells

#### 2.3.1. Cancer-Associated Fibroblasts

Cancer-associated fibroblasts (CAFs) are one of the most abundant nonimmune cells present in the TME. CAFs modulate cancer metastasis via secretion of several growth factors and cytokines, as well as by remodeling the extracellular matrix (ECM) and influencing angiogenesis. Moreover, evidence suggests that CAFs contribute to therapy resistance through different mechanisms, such as via cell–matrix and cell–cell interactions that control cell survival and through paracrine signaling to cancer cells upon the secretion of pro-survival factors and a variety of modulatory molecules like TGF-β [54]. The metabolism of CAFs is also implicated in their protumoral activity. In the so-called reverse Warburg effect, cancer cells reprogram the metabolism of neighboring CAFs to adopt aerobic glycolysis, partly through ROS-induced oxidative stress. These CAFs secrete high amounts of pyruvate and lactate, which are taken up by cancer cells to support their metabolic needs [55]. Moreover, increased expression of glutamine synthetase (GS) and higher glutamine secretion by CAFs can also support tumor growth, as seen, for instance, in pancreatic and ovarian cancer cells [56,57]. Importantly, in an orthotopic mouse model of ovarian carcinoma, co-targeting GS in CAFs and glutaminase in cancer cells effectively reduced tumor growth and metastasis [57], which underscores the significance of targeting the stromal cancer metabolic crosstalk.

#### 2.3.2. Tumor Endothelial Cells

The TME is characterized by morphologically and functionally abnormal vessel networks. Strong evidence suggests that tumor endothelial cells (TECs) have different phenotypic and functional features when compared to normal endothelial cells (NECs). Since TECs are highly proliferative and present increased potential for self-renewal, they display a relevant functional role as promoters of tumor angiogenesis. Simultaneously, TECs are mediators of immune regulation in the TME, acting as antigen-presenting cells, associated with T-cell priming, activation, and proliferation [56]. By transcriptomic and metabolomic analysis, Cantelmo and colleagues showed that TECs have a hyperglycolytic metabolism [57]. A metabolic pathway analysis revealed that the glycolytic flux was three-fold higher in TECs compared to NECs, while the glucose oxidation and oxygen consumption associated with ATP production were not altered. Moreover, inhibition of the glycolytic activator PFKFB3 (6-phosphofructo-2-kinase/fructose-2,6-bisphosphatase 3) proved to be effective in inducing tumor vessel normalization, reducing metastasis and improving chemotherapy [57,58]. In addition to glycolysis, mitochondrial respiration has also been attributed a crucial role in endothelial cells, with mitochondrial complex III deemed essential for their proliferation during angiogenesis [59]. Further metabolic requirements associated with vessel sprouting include glutaminolysis [60] and fatty acid oxidation (FAO) [61].

#### 2.3.3. Cancer-Associated Adipocytes

In solid tumors that grow in the vicinity of adipose tissue, the TME comprises relevant amounts of adipocytes, which, upon interaction with cancer cells, acquire molecular features that promote tumor growth and invasion. These so-called cancer-associated adipocytes (CAA) are postulated to influence tumor development via multiple mechanisms, including the release of adipokines, growth factors and hormones, the recruitment of immune cells, the production of proteases that degrade the ECM and facilitate cell migration, and the metabolic reprogramming of cancer cells [62]. Several studies based on cocultures of adipocytes and cancer cells demonstrate the establishment of a symbiotic metabolic relationship, whereby adipocyte lipolysis and lipid release, stimulated by cancer cells, serve to supply cancer cells with fatty acids, increasing their reliance on β-oxidation for energy production [63,64]. Additionally, a recent study has shown that naïve adipocytes, unstimulated by tumor cells, can also provide lipids to melanoma cells through extracellular vesicles, found to contain not only lipids but also the enzymatic machinery needed for FAO [65]. Hence, targeting lipolysis in adipocytes and their metabolic crosstalk with cancer cells could represent an additional strategy to improve anticancer therapies. 

Overall, the main phenotypic and metabolic features of cells in the tumor microenvironment are summarized in Table 1.

## 3. Metabolic Modulation of TME Cells by Natural Compounds

### 3.1. Curcumin

**Figure 1:**
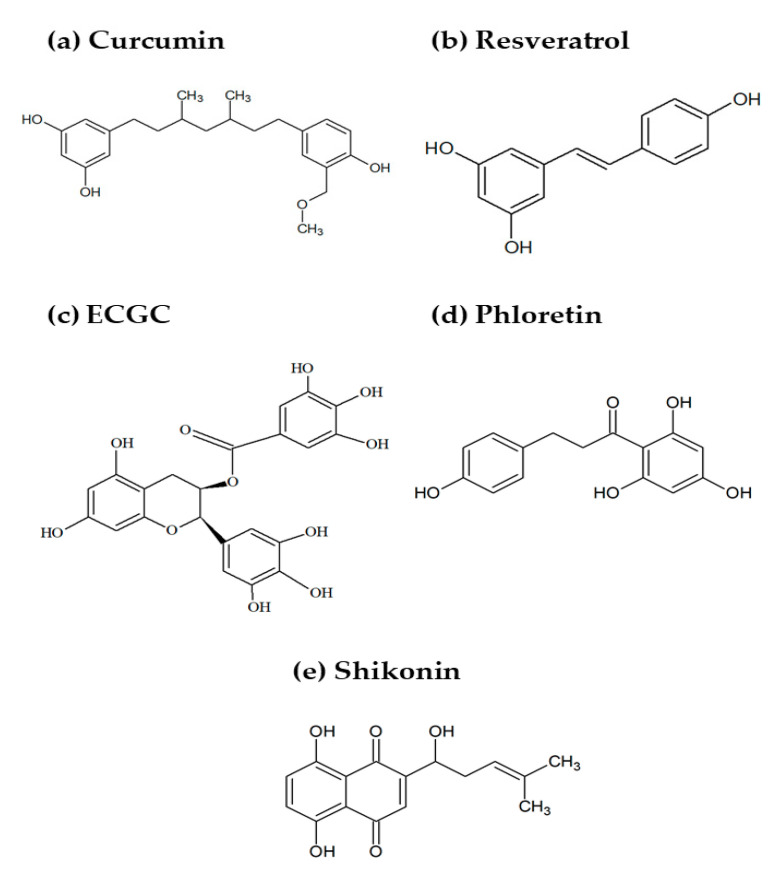
Chemical structures of some natural compounds reported to alter the metabolism of stromal cells in the TME: (**a**) curcumin, (**b**) resveratrol, (**c**) epigallocatechin gallate (EGCG), (**d**) phloretin, and (**e**) shikonin.

Curcumin (Figure 1a) is a hydroxycinnamic acid present in the rhizome of *Curcuma longa* (turmeric), which is used as a dietary spice. It has potent antiproliferative and proapoptotic effects in tumor cells of various origins, and it can alter their susceptibility to radio- or chemotherapy treatments [68], as well as to anticancer gene therapy [69]. Moreover, curcumin can inhibit the replication and/or reactivation of herpesvirus involved in the etiology of human cancers, such as Kaposi’s sarcoma-associated herpesvirus (KSHV) and the Epstein–Barr virus (EBV) [70]. The activities of curcumin in tumor cells involve multiple signaling pathways and molecular targets, including inflammatory mediators; transcription factors, growth factors; and proteins orchestrating cell survival, proliferation, and death. In recent years, curcumin’s antitumoral action has also been linked to its metabolic effects [71]. Subtoxic levels of curcumin inhibited glucose uptake and glycolytic conversion to lactate in several cancer cell lines by decreasing the expression of key glycolytic enzymes like hexokinase 2 (HK2) [72] and pyruvate kinase isoform M2 (PKM2) [73,74]. As many cancer cells strongly depend on the Warburg metabolism for rapid energy production and macromolecular synthesis, this effect may contribute to curcumin’s antiproliferative activity. Importantly, glycolysis inhibition and reduced extracellular lactate levels were accompanied by the downregulation of lactate hydroxycarboxylic acid receptor-1 (HCAR-1/GPR81) in hepatic carcinoma cells. As HCAR-1 modulates the multidrug resistance (MDR) protein family involved in cytotoxic drug expelling, this could explain the curcumin-induced sensitization to chemotherapy drugs [75]. Moreover, the antitumoral effects of curcumin were related to inhibition of fatty acid synthase (FASN) [74,76,77], a key enzyme in de novo lipid synthesis, as well as to its ability to inhibit ATP synthase activity, resulting in impaired mitochondrial respiration, increased production of ROS, and apoptosis [78].

Curcumin has also been shown to target CSCs and cause their elimination through interference with several biological processes and pathways [79]. At the metabolic level, curcumin (40 µM, 48 h) was proposed to interfere with glutamine uptake in colon CSCs, possibly via direct coupling with CD44 at the cell surface [80]. In that study, purported CSCs were isolated from the HT29 colorectal cancer cell line through CD44-positive selection using magnetic beads. A 48h-treatment with 50 µM of curcumin induced apoptosis in CD44^+^ cells but not in CD44^−^ cancer cells, suggesting that curcumin preferentially targets the CSC subpopulation within colorectal cancer cells. Through mass spectrometry-based metabolic profiling, curcumin was also found to differentially affect the metabolism of CD44^+^ and CD44^−^ cells. The former showed significantly reduced intracellular levels of glutamine, an amino acid that typically serves as an anaplerotic substrate to sustain the increased energetic needs of cancer cells. Based on the unchanged ATP levels observed in curcumin-treated CD44^+^ cells (which excluded intensified OXPHOS), the authors hypothesized that glutamine uptake could be blocked due to direct interaction of curcumin with CD44 at the cell membrane and that this metabolic disruption could induce apoptosis of CSCs. On the other hand, corroborating this hypothesis, the glutamine levels were not affected in curcumin-treated CD44^−^ cancer cells [80].

Regarding stromal TME cells, curcumin was found to modulate the lipid metabolism in THP-1-derived macrophages. In particular, it induced lipid accumulation by upregulating the expression of lipid transport genes, such as fatty-acid transporter (CD36/FAT) and fatty acid-binding protein-4 (FABP-4) [81,82]. The authors suggested that lipid accumulation in macrophages could be a mechanism through which curcumin helps lowering the lipid levels in the bloodstream. In the context of the TME, this may reduce the availability of the lipids for cancer cells, which, in turn, could contribute to impairing the tumor growth.

### 3.2. Resveratrol

Resveratrol (Figure 1b) is a stilbenoid produced by many plants in response to stress factors and is commonly found in the skin of grapes, berries, and peanuts. Among other biological activities (e.g., anti-inflammatory, antioxidant, and cardioprotective), the chemopreventive and anticancer effects of resveratrol have been widely reported and reviewed [83,84]. Like curcumin, the antitumoral activity of resveratrol is multitargeted and comprises interference with the cell cycle and death mechanisms of tumor cells, together with the modulation of the processes involved in oncogenic signaling in the tumor microenvironment, such as hypoxia, oxidative stress, and inflammation.

Resveratrol rewires glucose metabolism of tumor cells by inhibiting glycolysis and upregulating OXPHOS, in association with PKM2 downregulation and AMPK activation, as reviewed in reference [85]. Notably, this ability to shift the glycolytic-to-oxidative balance of tumor cells was recently shown to enhance the antitumor effect of silencing PD-L1 (programmed cell death protein ligand 1), a protein that hinders the cytotoxic activity of T cells [86]. In that study, resveratrol (10 µM) was co-delivered with PD-L1 siRNA, through copolymer-based polyplexes and found to stimulate mitochondrial OXPHOS while downregulating the glycolytic enzymes and lactate production in melanoma (B16F10) and colorectal (CT26) cancer cell lines. These effects were also observed in vivo, after the injection of resveratrol-containing polyplexes into tumor mouse models. The abrogation of glycolysis and consequent decrease in tissue lactic acidosis could, in itself, be expected to mitigate the immunosuppressive TME [7]. However, the accompanying upregulation of mitochondrial respiration was found key to enhance the immune responses [86], consisting of higher infiltration of CD8^+^ and CD4^+^ T cells, the inhibition of Tregs and myeloid derived suppressor cells (MDSCs), and increased secretion of interferon-gamma (IFN-γ), a cytokine that stimulates Th1 responses and macrophage activation and, thus, promotes antitumoral immunity.

The resveratrol-induced stimulation of mitochondrial OXPHOS was also reported in nasopharyngeal carcinoma (NPC) CSCs [87]. CSCs isolated serially from NPC cell lines through irradiation, sphere formation, and side population selection displayed a more glycolytic phenotype compared to parental cells, characterized by lower oxygen consumption and higher lactate secretion. On the other hand, the treatment with resveratrol (50 µM) opposed these changes and stimulated OXPHOS in CSCs, as corroborated by the downregulation of pyruvate dehydrogenase kinase (PDK), a suppressor of pyruvate dehydrogenase (PDH), which results in shifting pyruvate back to mitochondrial metabolism. Additionally, resveratrol induced higher ROS levels and mitochondrial membrane depolarization in NPC CSCs. Mechanistically, induction of the tumor suppressor p53 was proposed to be at the basis of resveratrol-induced changes in CSCs properties, as the overexpression and silencing of this gene produced clear responses in terms of CSCs stemness, the EMT, and metabolic reprogramming [87]. 

The importance of resveratrol-mediated metabolic reprogramming in the TME was further demonstrated by its direct effects on T cells [88]. The exposure of human CD4^+^ T cells (isolated from peripheral blood of healthy donors) to low-dose resveratrol (20 µM) downregulated the membrane glucose transporter 1 (GLUT1) and decreased the glucose uptake and glycolysis, as monitored by the production of lactate and extracellular acidification. Moreover, resveratrol induced a higher glutamine consumption by lymphocytes, as shown by increased glutamine transporter ASCT2, previously found to be critical for T-cell activation [89]. This was accompanied by an upregulation of glutaminase 2 (GLS2), an enzyme that catalyzes the conversion of glutamine into glutamate, and by an increased glutamine uptake, all data indicating the resveratrol-induced stimulation of glutaminolysis in T cells. Moreover, resveratrol-treated lymphocytes displayed an increased oxygen consumption rate (OCR), which indicates a shift to OXPHOS, corroborated by increased intracellular ATP levels and a higher production of mitochondrial ROS. These metabolic changes were linked to the activation of p53, which was mediated by a genotoxic stress response involving kinase ataxia telangiectasia-mutated and Rad3-related ATR. Most importantly, the enhancement of T-cell bioenergetic fitness by resveratrol was associated with increased IFN-γ secretion and, thus, an augmented effector function.

### 3.3. Epigallocatechin Gallate

Epigallocatechin gallate (EGCG) (Figure 1c) is the most abundant and bioactive catechin in green tea, and its anticancer effects have been extensively studied. As recently reviewed [90], EGCG can hit a variety of molecular targets in different cancer cells and induce antiproliferative, antioxidant, anti-inflammatory, and antiangiogenic effects at all stages of carcinogenesis. A few studies have additionally shown that EGCG interferes with tumor cell metabolism [91,92]. In breast cancer cells, concomitantly with the induction of autophagy and apoptosis, EGCG (20–240 µM) downregulated the expression of the glycolytic regulators GLUT1 and hypoxia-inducible factor 1-α (HIF-1α) and inhibited several glycolytic enzymes, thus hampering the glucose metabolism [91]. Moreover, in colon cancer cells, this flavonoid (50 µg/mL) was shown by joint transcriptomics and metabolomics analyses to impact other metabolic pathways, namely glycerophospholipid metabolism and glutathione metabolism, likely related to antiproliferative and antioxidant actions, respectively [92]. The metabolic effects underlying the ability of a green tea extract (GTE) and EGCG (50 µM) to inhibit the proliferation of umbilical vein endothelial cells (HUVECs), thus avoiding neovascularization, have also been recently described [93]. GTE was found to downregulate the pathways related to the synthesis of cellular building blocks (nucleotides, nucleotide sugars, amino acids, and pantothenic acid); mitochondrial energy production; and inositol signaling, all postulated to explain GTE’s antiproliferative actions. On the other hand, it triggered protective mechanisms by activating the pathways related to vitamin B6, glycerophospholipids, and antioxidants production, thus maintaining the cellular integrity. Interestingly, EGCG also exerted inhibitory and protective effects but through different pathways, as revealed by metabolic profiling. Growth inhibition was ascribed to prooxidant effects and the suppression of membrane signaling molecules, while cellular protection appeared to be promoted via the upregulated expression of vitamins B6 and B2, NAD, and putrescine. Comparatively to catechins, the antiangiogenic drug Bevacizumab, which blocks proliferation by specifically inhibiting the binding of vascular endothelial growth factor (VEGF) to its receptor, displayed a narrower spectrum of metabolic effects. It mainly suppressed the biosynthesis of amino acids and increased polyunsaturated fatty acids expression, which may alter the membrane properties and affect cellular proliferation. Altogether, the multitargeting activity of GTE and catechin mixtures in endothelial cells, which could be largely explained at the metabolic level, represents a valuable feature in antiangiogenic approaches.

### 3.4. Phloretin

Phloretin (Figure 1d) is a hydroxylated dihydrochalcone present in the root bark and leaves of apple and other fruit trees. It was shown to inhibit glucose transporters in breast [94] and colon cancer cells [95], an effect that has been related to cell growth suppression. Recently, its potential role in modulating the TME metabolism, namely the tumor–fibroblasts metabolic crosstalk, has been highlighted [96]. To induce a CAF-like state, bone marrow-derived mesenchymal stem cells (MSCs), at 60–70% confluence, were incubated for up to 30 days in a medium conditioned by MDA-MB-231 breast cancer cells. The CAFs were shown to oxidize lactate into pyruvate, which, in turn, supported the biosynthetic, energetic, and antioxidant needs of cancer cells. This lactate–pyruvate metabolic loop was disrupted by phloretin (100 µM), which significantly attenuated glycolysis and ROS accumulation in cancer cells, while disrupting the lactate uptake in CAFs. In addition, phloretin enhanced the cytotoxicity of doxorubicin (a conventional chemotherapy drug) in the presence of a CAF-conditioned medium, but it did not contribute to drug cytotoxicity in the complete medium. Overall, phloretin was demonstrated to be a powerful adjuvant to potentiate the effects of anticancer drugs, due to its efficacy in downregulating the glucose uptake and monocarboxylate exchange, which are key metabolic dependencies of tumors [96].

### 3.5. Shikonin

Shikonin (Figure 1e) is a naturally occurring naphthoquinone found in the root of plants from the Boraginaceae family and the first compound to be obtained from large-scale plant cell cultures [97]. It has been used in traditional Chinese medicine for centuries and shown to possess several therapeutic properties, including antimicrobial, wound healing, anti-inflammatory, antioxidant, and anticancer activities [98]. The potential of shikonin and its derivatives in cancer treatment has received increasing attention in recent years, mainly due to its wide spectrum antitumor effects [99]. The repression of glycolysis through the specific inhibition of PKM2, the enzyme catalyzing the conversion of phosphoenolpyruvate to pyruvate, is a key mechanism in shikonin’s antitumor activity, as demonstrated in a variety of tumor cells [100,101,102]. Moreover, recent data clearly showed that shikonin-mediated metabolic effects impacted the TME by repolarizing TAM and synergizing with PD-1 blockage (mediated by JQ1), thus enhancing the immune response [103]. In that work, mannosylated lactoferrin nanoparticles were loaded with shikonin (1 µM) and JQ1 (3 µM) for a targeted codelivery to colon cancer cells (CT26) and TAM. The bioactive NPs reduced lactate production in cancer cells (in association with PKM2-mediated glycolytic inhibition) and skewed macrophages towards a proinflammatory phenotype, characterized by a higher production of TNF-α and lower secretion of TGF-β. Furthermore, the treatment of CT26 tumor-bearing mice with the shikonin/JQ1-loaded nanosystem efficiently decreased the tumor growth; suppressed the glucose metabolism (as seen by reduced levels of lactate, PKM2, and HIF-1α in the tumor tissue); downregulated the intratumoral PD-L1 expression; and remodeled the TME’s immune configuration (e.g., the promotion of dendritic cell maturation and CD8^+^ T-cell infiltration, as well as suppression of Treg). Overall, the synergism between metabolic reprogramming and the regulation of immune responses was shown to improve the antitumor treatment efficacy.

### 3.6. Other Natural Compounds

Ribosomally synthesized and post-translationally modified peptides (RiPP) are a group of microbial natural products that are attracting increasing attention as novel antitumor drugs [104]. One of these compounds, thioholgamide (thioA), identified as a product of *Streptomyces* sp., was recently shown to exhibit dual anticancer activity by targeting both cancer cells and tumor-associated macrophages [105]. Even at subtoxic nanomolar concentrations, thioA was found to act on the metabolism and migration of liver (RIL175) and/or colon (HCT116) carcinoma cells, demonstrating its ability to modulate key cancer hallmarks. Additionally, thioA skewed in vitro differentiated human monocyte-derived macrophages (HMDMs) polarized to M2- or TAM-like phenotypes through incubation with interleukin-4/13 (IL-4/IL-13) or a tumor-conditioned medium, respectively, to a more antitumoral profile. This profile included the downregulation of OXPHOS-dependent energy production and a compensatory glycolytic increase, together with reduced phagocytic capacity and downregulation of anti-inflammatory polarization markers (IL-10 and CD163) and upregulation of the proinflammatory cytokine interferon-gamma-inducible protein 10 (IP-10). 

The phenotypic and bioenergetic reprogramming of tumor-supporting M2-like macrophages to tumoricidal M1-like macrophages was also achieved with yeast-derived particulate β-glucan [106]. Mouse bone marrow-derived macrophages (BMDMs) pre-polarized to M2 with macrophage colony-stimulating factor (M-CSF) or IL-4/IL-13 responded to this natural polysaccharide by downregulating the mRNA expression of several conventional M2 marker genes (such as IL-10 and arginase I) while promoting the M1 gene expression signature (including iNOS, IL-12p35, TNF-α, IL-1β, and IL-6). Concomitantly, β-glucan (100 µg/mL) induced the accumulation of arginine, consistent with the suppression of arginase, and enhanced glycolysis, the TCA cycle, and glutamine utilization. These metabolic events, unveiled through stable isotope resolved metabolomics (SIRM), were largely coincident with those triggered by GM-CSF M1 polarization, which underscores their importance in the β-glucan-mediated polarization shift. Importantly, the β-glucan treatment also repolarized immunosuppressive TAM isolated from murine tumor models, both phenotypically and functionally (by promoting T-cell responses), and reduced the tumor burden in vivo. 

Another recent study reported the ability of two flavonoid derivatives to oppose the effects induced by hemin in THP-1-derived macrophages [107]. Hemin is a myoglobin-derived metabolite related to meat intake, which promotes an anti-inflammatory phenotype in macrophages via the activation of heme-oxygenase (HO)-1 [108]. According to reference [107], the exposure to hemin during the stimulation with lipopolysaccharide (LPS) and IFN-γ led macrophages to produce lower levels of IP-10, prevented the upregulation of glycolysis (as assessed by the activity of some glycolytic enzymes and measurement of extracellular acidification), and diminished the macrophage capacity to engulf and kill A375 melanoma cells. Notably, the flavonoid derivatives 3,4-dihydroxyphenylacetic acid (3,4DHPAA) and 4-hydroxyphenylacetic acid (4HPAA) were able to largely counteract the hemin-induced effects. These metabolites originate from the microbial transformation of major dietary phenolics, namely quercetin and its glycosylated compounds in the case of 3,4DHPAA and proanthocyanidins and kaempferol in the case of 4HPAA. Treating THP-1-derived macrophages with noncytotoxic concentrations of these compounds (10 µM) during the 72h-exposure to hemin (10 µM) rescued the IP-10 production and upregulated glycolytic activity and several metabolic enzymes involved in glycolysis and the PPP. Additionally, it stabilized HIF-1α, a positive regulator of glycolysis, as evidenced by the increased cytosolic protein levels. Moreover, both phenolic derivatives rescued the capacity of activated macrophages to engulf and kill cocultured cancer cells, an outcome likely associated to macrophage metabolic reprogramming.

Some phytochemicals have also been credited for suppressing adipocyte differentiation and inhibiting adipogenic activity. For instance, EGCG and apigenin (a flavone present in many plants) inhibited the differentiation of preadipocytes (3T3-L1) to mature adipocytes, significantly decreasing the lipid accumulation in these cells [109,110]. Mechanistically, apigenin acted by reducing the expression of CD36 and peroxisome proliferator-activated receptor gamma (PPAR-γ) via the upstream downregulation of the signal transducer and activator of transcription 3 (STAT3), a promoter of adipogenesis whose expression is upregulated during adipocyte differentiation [110]. The triterpene maslinic acid (MA) also had an antiadipogenic effect in 3T3-L1 cells, which resulted in a decreased lipid synthesis and enhanced glucose uptake, possibly as an alternative energetic fuel to adipocytes [111]. The antiadipogenic effect of these and other natural compounds has been highlighted in the context of anti-obesity therapy. However, given the protumoral role of adipocytes in providing fatty acids to neighbor cancer cells in the TME [112], the antiadipogenic activity of natural compounds could also be beneficial in the context of cancer treatment. This is even more so if a glucose uptake is also stimulated, as seen with MA, since this could potentially limit the glucose availability to cancer cells.

## 4. Concluding Remarks 

The improved understanding of the central role of the TME in cancer development, progression, and response to treatment has opened a plethora of new perspectives on how modulating the complex tumor milieu, rather than targeting only malignant cells, could make a difference in therapeutic approaches. The use of bioactive natural compounds for this purpose has attracted increasing interest, as many of them have multiple biological activities beyond antiproliferative and proapoptotic effects in tumor cells that can potentially be harnessed to promote an antitumoral microenvironment. 

**Figure 2:**
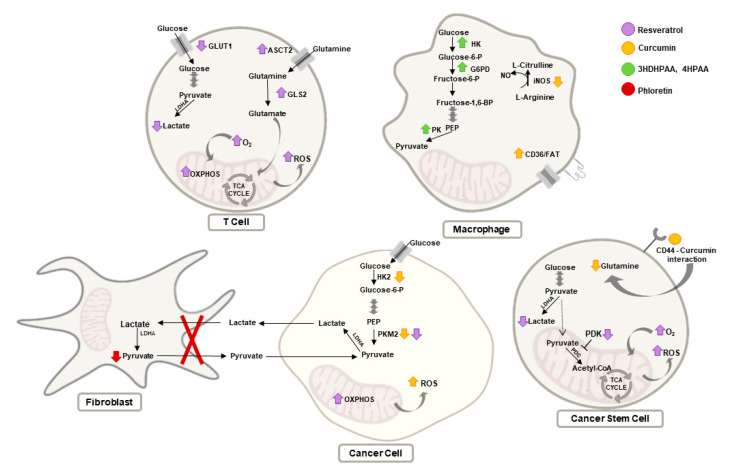
Overview of the main effects of the selected natural compounds on the metabolism of cells present in the TME, namely T cells, macrophages, cancer, and cancer stem cells, as well as on the disruption of the crosstalk between fibroblasts and cancer cells. Metabolic alterations induced by resveratrol are represented in purple, by curcumin in yellow, by the flavonoid derivatives 3,4DHPAA and 4HPAA in green, and by phloretin in red. Abbreviations: 3,4DHPAA, 3,4-dihydroxyphenylacetic acid; 4HPAA, 4-hydroxyphenylacetic acid; Acetyl-CoA, acetyl-coenzyme A; ASCT2, alanine-serine-cysteine transporter 2; FAT, fatty acid transporter; Fructose-1,6-BP, fructose 1,6-bisphosphate; Fructose-6-P, fructose-6-phosphate; G6PD, glucose-6-phosphate dehydrogenase; GLS2, glutaminase 2; Glucose-6-P, glucose-6-phosphate; GLUT1, glucose transporter 1; HK, hexokinase; LDHA, lactate dehydrogenase A; PDK, pyruvate dehydrogenase kinase; PEP, phosphoenolpyruvate; PHD, pyruvate dehydrogenase complex; PK, pyruvate kinase; ROS, reactive oxygen species; TCA cycle, tricarboxylic acid cycle.

The rewiring of cellular metabolism is an emergent TME-modulating strategy, and several natural compounds have shown promise in this respect, as highlighted by the illustrative examples discussed in this review (Figure 2). A common feature to several of these compounds is their negative regulation of glycolysis in cancer cells and consequent decrease of lactate secretion, which can have important implications in shaping the TME. This has been clearly shown for phloretin, which disrupted the lactate–pyruvate loop between CAF and cancer cells, and for the shikonin-induced repression of glucose metabolism, which influenced TAM polarization and contributed to remodeling the immune component of the TME.

Additionally, some natural compounds (thioA, β-glucan, and flavonoid derivatives) could stimulate glycolysis in macrophages and favor their antitumoral role, while others could limit the availability of lipids to cancer cells by hindering adipogenesis in TME adipocytes (apigenin and maslinic acid) or promoting their accumulation in macrophages (curcumin). Furthermore, the metabolic effects induced by EGCG in endothelial cells and by resveratrol in T cells, related, respectively, to the antiangiogenic and antitumor immune effector functions, are relevant examples of the far-reaching and multitargeted activity of natural compounds as anticancer metabolic modulators.

There is still much to discover about the impact of natural compounds on the metabolism of TME cells and their crosstalk. By providing a holistic picture of effects, omics approaches, particularly proteomics and metabolomics, represent valuable tools in this respect, and their integration in biological studies should help in disclosing the intricate aspects of TME metabolism and its modulation. The development of adequate and more realistic cellular models is another critical challenge in TME studies. Advances in 3D culture platforms, such as heterotypic spheroids, organoids, and microfluidic cancer-on-a-chip models [113], will certainly enable a better recapitulation of the TME and its dynamic changes. Finally, the results of cancer clinical trials performed with natural compounds such as curcumin, resveratrol, catechins, and shikonin, listed, respectively, in references [68,85,90,99], should be carefully considered. Such trials often reveal that the clinical application of these molecules may be severely limited by issues like poor solubility, rapid biotransformation upon oral administration, reduced bioavailability and tissue distribution, and/or unacceptable toxicity to normal cells. Several strategies have been devised to overcome these limitations, including the combination with other bioactive compounds, chemical modification to increase stability and/or water solubility, and inclusion in carrier nanosystems. There are indeed many examples of the successful nanoencapsulation of phytochemicals, which protects them from degradation, improves their pharmacokinetic profile, and/or enables the selective delivery to specific cell types/tissues, thus avoiding unwanted side effects [114]. Moreover, nanoencapsulation offers the possibility to combine different bioactive agents to achieve multiple, synergistic effects, as recently illustrated by the combination of anti-PD-L1 therapeutics with resveratrol [86] or shikonin [86] in copolymer-based polyplexes and mannopyranoside-lactoferrin nanoparticles, respectively. Overall, it is clear that the pleiotropic anticancer potential of a panoply of natural compounds should be further explored in both preclinical and clinical studies to advance the current understanding of their mechanisms of action in the complex TME, as well as to potentiate the effects that will result in improved safety and efficacy profiles in vivo.

## Figures and Tables

**Table 1:** Main phenotypic and metabolic features of cells in the tumor microenvironment.

Cells in the TME	Phenotypic Features	Metabolic Features	Ref.
**Malignant Cells**			
Cancer Cells	Proangiogenic Invasion and metastasis Immune evasion Immunosuppression	↑ Aerobic glycolysis (lactate secretion) ↑ Glutaminolysis ↑ PPP ↑ One-carbon metabolism ↑ de novo lipid synthesis	[7,17,18]
Cancer Stem Cells (CSCs)	Expression of surface markers (CD44, CD133 or ALDH1) Stemness potential Prometastatic Protumorigenic Resistance to chemo/-radiation	Mitochondrial respiration (↑ mitochondrial mass, ↑ oxygen consumption)	[19,20,21,22,23]
**Immune Cells**			
Tumor-Associated Macrophages	M1-like phenotype: - Proinflammatory - Tumoricidal functions	↑ Glycolysis Citrate and succinate accumulation	[27,28,29,30,31,32,33]
	M2-like phenotype: - Anti-inflammatory - Protumorigenic - Positive correlation with poor prognosis in cancer patients	↑ TCA cycle and OXPHOS Itaconate production	
T Lymphocytes	Cytotoxic T cells (Tc): - Antitumoral activities	Aerobic glycolysis (↑ glucose uptake, ↑ PPP, ↑ glutamine metabolism)	[38,39,40,41,42,43]
	Regulatory T cells (Treg): - Inhibition of Tc activity - Positive correlation with poor prognosis in cancer patients	↑ OXPHOS	
Natural Killer Cells (NK cells)	Antitumoral activity Positive correlation with favorable prognosis in cancer patients	↑ Glycolysis and OXPHOS to enhance cytotoxic capacity	[44,45,46,47]
Dendritic Cells (DCs)	Mature/activated DCs: - Activation of T lymphocytes - Induction of adaptive immune response	↑ Glycolysis during activation	[48,49,50,51,52,53]
	Tolerogenic DCs: - Inhibition of immune response - ↑ Expression of IDO - Promotion of Treg - Suppression of Tc	↑ TCA cycle	
**Nonimmune Stromal Cells**			
Cancer-Associated Fibroblasts (CAFs)	Protumoral activity: - Modulation of cancer metastasis - ECM remodeling - Therapy resistance	Reverse Warburg effect (aerobic glycolysis) ↑ Glutamate and glutamine secretion (↑ glutamine synthetase)	[54,55,66,67]
Tumor Endothelial Cells (TECs)	Promotion of angiogenesis: - Highly proliferative - Self-renewal potential Can act as APC	↑ Glycolysis ↑ Mitochondrial respiration (mitochondrial complex III), glutaminolysis and FAO	[56,57,58,59]
Cancer-Associated Adipocytes (CAA)	Promotion of tumor growth and invasion: - Release of adipokines, growth factors and hormones - Recruitment of immune cells - Production of proteases	↑ Lipolysis and lipid release	[62,63,64,65]

Abbreviations: ALDH1, aldehyde dehydrogenase 1; APC, antigen presenting cells; ECM, extracellular matrix; FAO, fatty acid oxidation; IDO, indoleamine 2,3-dioxygenase; OXPHOS, oxidative phosphorylation; PPP, pentose phosphate pathway; TCA cycle, tricarboxylic acid cycle.

## References

[1] Labani-Motlagh A., Ashja-Mahdavi M., Loskog A. (2020). The Tumor Microenvironment: A Milieu Hindering and Obstructing Antitumor Immune Responses. Front. Immunol..

[2] Wu T., Dai Y. (2017). Tumor microenvironment and therapeutic response. Cancer Lett..

[3] Hanahan D., Weinberg R.A. (2011). Hallmarks of Cancer: The Next Generation. Cell.

[4] Fouad Y.A., Aanei C. (2017). Revisiting the hallmarks of cancer. Am. J. Cancer Res..

[5] Romero-Garcia S., Lopez-Gonzalez J.S., Báez-Viveros J.L., Aguilar-Cazares D., Prado-Garcia H. (2011). Tumor cell metabolism: An integral view. Cancer Biol. Ther..

[6] Dias A.S., Almeida C.R., Helguero L., Duarte I.F. (2019). Metabolic crosstalk in the breast cancer microenvironment. Eur. J. Cancer.

[7] De La Cruz-López K.G., Castro-Muñoz L.J., Reyes-Hernández D.O., García-Carrancá A., Manzo-Merino J. (2019). Lactate in the Regulation of Tumor Microenvironment and Therapeutic Approaches. Front. Oncol..

[8] Xing Y., Zhao S., Zhou B.P., Mi J. (2015). Metabolic reprogramming of the tumour microenvironment. FEBS J..

[9] Zyad A., Leouifoudi I., Tilaoui M., Mouse H.A., Khouchani M., Jaafari A. (2018). Natural Products as Cytotoxic Agents in Chemotherapy against Cancer. Cytotoxicity.

[10] Liskova A., Samec M., Koklesova L., Brockmueller A., Zhai K., Abdellatif B., Siddiqui M., Biringer K., Kudela E., Pec M. (2021). Flavonoids as an effective sensitizer for anti-cancer therapy: Insights into multi-faceted mechanisms and applicability towards individualized patient profiles. EPMA J..

[11] Park S., Surh Y. (2017). Modulation of tumor microenvironment by chemopreventive natural products. Ann. N. Y. Acad. Sci..

[12] Zubair H., Khan M.A., Anand S., Srivastava S.K., Singh S., Singh A.P. (2020). Modulation of the tumor microenvironment by natural agents: Implications for cancer prevention and therapy. Semin. Cancer Biol..

[13] Pan P., Huang Y.-W., Oshima K., Yearsley M., Zhang J., Arnold M., Yu J., Wang L.-S. (2019). The immunomodulatory potential of natural compounds in tumor-bearing mice and humans. Crit. Rev. Food Sci. Nutr..

[14] Focaccetti C., Izzi V., Benvenuto M., Fazi S., Ciuffa S., Giganti M.G., Potenza V., Manzari V., Modesti A., Bei R. (2019). Polyphenols as Immunomodulatory Compounds in the Tumor Microenvironment: Friends or Foes?. Int. J. Mol. Sci..

[15] Samec M., Liskova A., Koklesova L., Samuel S.M., Zhai K., Buhrmann C., Varghese E., Abotaleb M., Qaradakhi T., Zulli A. (2020). Flavonoids against the Warburg phenotype—concepts of predictive, preventive and personalised medicine to cut the Gordian knot of cancer cell metabolism. EPMA J..

[16] Guerra A.R., Duarte M.F., Duarte I.F. (2018). Targeting Tumor Metabolism with Plant-Derived Natural Products: Emerging Trends in Cancer Therapy. J. Agric. Food Chem..

[17] DeBerardinis R.J., Chandel N.S. (2016). Fundamentals of cancer metabolism. Sci. Adv..

[18] Heiden M.G.V., DeBerardinis R.J. (2017). Understanding the Intersections between Metabolism and Cancer Biology. Cell.

[19] Shen Y.-A., Pan S.-C., Chu I., Lai R.-Y., Wei Y.-H. (2020). Targeting cancer stem cells from a metabolic perspective. Exp. Biol. Med..

[20] Das M., Law S. (2018). Role of tumor microenvironment in cancer stem cell chemoresistance and recurrence. Int. J. Biochem. Cell Biol..

[21] Peiris-Pagès M., Martinez-Outschoorn U.E., Pestell R.G., Sotgia F., Lisanti M.P. (2016). Cancer stem cell metabolism. Breast Cancer Res..

[22] De Luca A., Fiorillo M., Peiris-Pagès M., Ozsvari B., Smith D.L., Sanchez-Alvarez R., Martinez-Outschoorn U.E., Cappello A.R., Pezzi V., Lisanti M.P. (2015). Mitochondrial biogenesis is required for the anchorage-independent survival and propagation of stem-like cancer cells. Oncotarget.

[23] Farnie G., Sotgia F., Lisanti M.P. (2015). High mitochondrial mass identifies a sub-population of stem-like cancer cells that are chemo-resistant. Oncotarget.

[24] Patil S. (2020). Metformin treatment decreases the expression of cancer stem cell marker CD44 and stemness related gene expression in primary oral cancer cells. Arch. Oral Biol..

[25] Song C.W., Lee H., Dings R., Williams B., Powers J., Dos Santos T., Choi B.-H., Park H.J. (2012). Metformin kills and radiosensitizes cancer cells and preferentially kills cancer stem cells. Sci. Rep..

[26] Cassetta L., Pollard J.W. (2018). Targeting macrophages: Therapeutic approaches in cancer. Nat. Rev. Drug Discov..

[27] Rhee I. (2016). Diverse macrophages polarization in tumor microenvironment. Arch. Pharmacal Res..

[28] Mantovani A., Sozzani S., Locati M., Allavena P., Sica A. (2002). Macrophage polarization: Tumor-associated macrophages as a paradigm for polarized M2 mononuclear phagocytes. Trends Immunol..

[29] Kurahara H., Shinchi H., Mataki Y., Maemura K., Noma H., Kubo F., Sakoda M., Ueno S., Natsugoe S., Takao S. (2011). Significance of M2-Polarized Tumor-Associated Macrophage in Pancreatic. Cancer J. Surg. Res..

[30] Lan C., Huang X., Lin S., Huang H., Cai Q., Wan T., Lu J., Liu J. (2013). Expression of M2-Polarized Macrophages is Associated with Poor Prognosis for Advanced Epithelial Ovarian Cancer. Technol. Cancer Res. Treat..

[31] Haque A.S.M.R., Moriyama M., Kubota K., Ishiguro N., Sakamoto M., Chinju A., Mochizuki K., Sakamoto T., Kaneko N., Munemura R. (2019). CD206+ tumor-associated macrophages promote proliferation and invasion in oral squamous cell carcinoma via EGF production. Sci. Rep..

[32] Benner B., Scarberry L., Suarez-Kelly L.P., Duggan M.C., Campbell A.R., Smith E., Lapurga G., Jiang K., Butchar J.P., Tridandapani S. (2019). Generation of monocyte-derived tumor-associated macrophages using tumor-conditioned media provides a novel method to study tumor-associated macrophages in vitro. J. Immunother. Cancer.

[33] Weiss J.M., Davies L.C., Karwan M., Ileva L., Ozaki M., Cheng R.Y., Ridnour L.A., Annunziata C.M., Wink D.A., McVicar D.W. (2018). Itaconic acid mediates crosstalk between macrophage metabolism and peritoneal tumors. J. Clin. Investig..

[34] Saha S., Shalova I.N., Biswas S.K. (2017). Metabolic regulation of macrophage phenotype and function. Immunol. Rev..

[35] Viola A., Munari F., Sánchez-Rodríguez R., Scolaro T., Castegna A. (2019). The Metabolic Signature of Macrophage Responses. Front. Immunology.

[36] Khan F.H., Dervan E., Bhattacharyya D.D., McAuliffe J.D., Miranda K.M., Glynn S.A. (2020). The Role of Nitric Oxide in Cancer: Master Regulator or NOt?. Int. J. Mol. Sci..

[37] Nath N., Kashfi K. (2020). Tumor associated macrophages and ‘NO’. Biochem. Pharmacol..

[38] Kouidhi S., Elgaaied A.B., Chouaib S. (2017). Impact of Metabolism in on T-Cell Differentiation and Function and Cross Talk with Tumor Microenvironment. Front. Immunol..

[39] Saleh R., Elkord E. (2020). FoxP3+ T regulatory cells in cancer: Prognostic biomarkers and therapeutic targets. Cancer Lett..

[40] Yin Z., Bai L., Li W., Zeng T., Tian H., Cui J. (2019). Targeting T cell metabolism in the tumor microenvironment: An anti-cancer therapeutic strategy. J. Exp. Clin. Cancer Res..

[41] Altman B.J., Dang C.V. (2012). Normal and cancer cell metabolism: Lymphocytes and lymphoma. FEBS J..

[42] Carr E.L., Kelman A., Wu G.S., Gopaul R., Senkevitch E., Aghvanyan A., Turay A.M., Frauwirth K.A. (2010). Glutamine Uptake and Metabolism Are Coordinately Regulated by ERK/MAPK during T Lymphocyte Activation. J. Immunol..

[43] Ho P.-C., Bihuniak J.D., Macintyre A., Staron M., Liu X., Amezquita R., Tsui Y.-C., Cui G., Micevic G., Perales J.C. (2015). Phosphoenolpyruvate Is a Metabolic Checkpoint of Anti-tumor T Cell Responses. Cell.

[44] Zhang S., Liu W., Hu B., Wang P., Lv X., Chen S., Shao Z. (2020). Prognostic Significance of Tumor-Infiltrating Natural Killer Cells in Solid Tumors: A Systematic Review and Meta-Analysis. Front. Immunol..

[45] Isaacson B., Mandelboim O. (2018). Sweet Killers: NK Cells Need Glycolysis to Kill Tumors. Cell Metab..

[46] Husain Z., Huang Y., Seth P., Sukhatme V.P. (2013). Tumor-Derived Lactate Modifies Antitumor Immune Response: Effect on Myeloid-Derived Suppressor Cells and NK Cells. J. Immunol..

[47] Harmon C., Robinson M.W., Hand F., AlMuaili D., Mentor K., Houlihan D.D., Hoti E., Lynch L., Geoghegan J., O’Farrelly C. (2018). Lactate-Mediated Acidification of Tumor Microenvironment Induces Apoptosis of Liver-Resident NK Cells in Colorectal Liver Metastasis. Cancer Immunol. Res..

[48] Plebanek M.P., Sturdivant M., DeVito N.C., Hanks B.A. (2020). Role of dendritic cell metabolic reprogramming in tumor immune evasion. Int. Immunol..

[49] Munn D.H., Sharma M.D., Lee J.R., Jhaver K.G., Johnson T.S., Keskin D.B., Marshall B., Chandler P., Antonia S.J., Burgess R. (2002). Potential Regulatory Function of Human Dendritic Cells Expressing Indoleamine 2,3-Dioxygenase. Science.

[50] Munn D.H., Mellor A.L. (2007). Indoleamine 2,3-dioxygenase and tumor-induced tolerance. J. Clin. Investig..

[51] Sharma M.D., Baban B., Chandler P., Hou D.-Y., Singh N., Yagita H., Azuma M., Blazar B.R., Mellor A.L., Munn D.H. (2007). Plasmacytoid dendritic cells from mouse tumor-draining lymph nodes directly activate mature Tregs via indoleamine 2,3-dioxygenase. J. Clin. Investig..

[52] O’Neill L.A., Pearce E.J. (2016). Immunometabolism governs dendritic cell and macrophage function. J. Exp. Med..

[53] Malinarich F., Duan K., Hamid R.A., Bijin A., Lin W.X., Poidinger M., Fairhurst A.-M., Connolly J.E. (2015). High Mitochondrial Respiration and Glycolytic Capacity Represent a Metabolic Phenotype of Human Tolerogenic Dendritic Cells. J. Immunol..

[54] Liao Z., Tan Z.W., Zhu P., Tan N.S. (2019). Cancer-associated fibroblasts in tumor microenvironment–Accomplices in tumor malignancy. Cell. Immunol..

[55] Karta J., Bossicard Y., Kotzamanis K., Dolznig H., Letellier E. (2021). Mapping the Metabolic Networks of Tumor Cells and Cancer-Associated Fibroblasts. Cells.

[56] Nagl L., Horvath L., Pircher A., Wolf D. (2020). Tumor Endothelial Cells (TECs) as Potential Immune Directors of the Tumor Microenvironment–New Findings and Future Perspectives. Front. Cell Dev. Biol..

[57] Cantelmo A.R., Conradi L.-C., Brajic A., Goveia J., Kalucka J., Pircher A., Chaturvedi P., Hol J., Thienpont B., Teuwen L.-A. (2016). Inhibition of the Glycolytic Activator PFKFB3 in Endothelium Induces Tumor Vessel Normalization, Impairs Metastasis, and Improves Chemotherapy. Cancer Cell.

[58] Zecchin A., Kalucka J., Dubois C., Carmeliet P. (2017). How Endothelial Cells Adapt Their Metabolism to Form Vessels in Tumors. Front. Immunol..

[59] Diebold L.P., Gil H.J., Gao P., Martinez C.A., Weinberg S., Chandel N.S. (2019). Mitochondrial complex III is necessary for endothelial cell proliferation during angiogenesis. Nat. Metab..

[60] Huang H., Vandekeere S., Kalucka J., Bierhansl L., Zecchin A., Brüning U., Visnagri A., Yuldasheva N.Y., Goveia J., Cruys B. (2017). Role of glutamine and interlinked asparagine metabolism in vessel formation. EMBO J..

[61] Schoors S., Bruning U., Missiaen R., Queiroz K.C., Borgers G., Elia I., Zecchin A., Cantelmo A.R., Christen S., Goveia J. (2015). Fatty Acid Carbon Is Essential for Dntp Synthesis in Endothelial Cells. Nature.

[62] Cao Y. (2019). Adipocyte and lipid metabolism in cancer drug resistance. J. Clin. Investig..

[63] Wen Y.-A., Xing X., Harris J.W., Zaytseva Y.Y., I Mitov M., Napier D.L., Weiss H.L., Evers B.M., Gao T. (2017). Adipocytes activate mitochondrial fatty acid oxidation and autophagy to promote tumor growth in colon cancer. Cell Death Dis..

[64] Wu Q., Li J., Li Z., Sun S., Zhu S., Wang L., Wu J., Yuan J., Zhang Y., Sun S. (2019). Exosomes from the tumour-adipocyte interplay stimulate beige/brown differentiation and reprogram metabolism in stromal adipocytes to promote tumour progression. J. Exp. Clin. Cancer Res..

[65] Clement E., Lazar I., Attané C., Carrié L., Dauvillier S., Ducoux-Petit M., Esteve D., Menneteau T., Moutahir M., Le Gonidec S. (2020). Adipocyte extracellular vesicles carry enzymes and fatty acids that stimulate mitochondrial metabolism and remodeling in tumor cells. EMBO J..

[66] Francescone R., Vendramini-Costa D.B., Franco-Barraza J., Wagner J., Muir A., Lau A.N., Gabitova L., Pazina T., Gupta S., Luong T. (2021). Netrin G1 Promotes Pancreatic Tumorigenesis through Cancer-Associated Fibroblast–Driven Nutritional Support and Immunosuppression. Cancer Discov..

[67] Yang L., Achreja A., Yeung T.-L., Mangala L.S., Jiang D., Han C., Baddour J., Marini J.C., Ni J., Nakahara R. (2016). Targeting Stromal Glutamine Synthetase in Tumors Disrupts Tumor Microenvironment-Regulated Cancer Cell Growth. Cell Metab..

[68] Giordano A., Tommonaro G. (2019). Curcumin and Cancer. Nutrients.

[69] Li H., Du H., Zhang G., Wu Y., Qiu P., Liu J., Guo J., Liu X., Sun L., Du B. (2019). Curcumin plays a synergistic role in combination with HSV-TK/GCV in inhibiting growth of murine B16 melanoma cells and melanoma xenografts. PeerJ.

[70] Šudomová M., Hassan S. (2021). Nutraceutical Curcumin with Promising Protection against Herpesvirus Infections and Their Associated Inflammation: Mechanisms and Pathways. Microorganisms.

[71] Vishvakarma N.K. (2014). Novel antitumor mechanisms of curcumin: Implication of altered tumor metabolism, reconstituted tumor microenvironment and augmented myelopoiesis. Phytochem. Rev..

[72] Wang K., Fan H., Chen Q., Ma G., Zhu M., Zhang X., Zhang Y., Yu J. (2015). Curcumin inhibits aerobic glycolysis and induces mitochondrial-mediated apoptosis through hexokinase II in human colorectal cancer cells in vitro. Anti-Cancer Drugs.

[73] Siddiqui F.A., Prakasam G., Chattopadhyay S., Rehman A.U., Padder R.A., Ansari M.A., Irshad R., Mangalhara K., Bamezai R.N.K., Husain M. (2018). Curcumin decreases Warburg effect in cancer cells by down-regulating pyruvate kinase M2 via mTOR-HIF1α inhibition. Sci. Rep..

[74] Yang R., Fang X.-L., Zhen Q., Chen Q.-Y., Feng C. (2019). Mitochondrial targeting nano-curcumin for attenuation on PKM2 and FASN. Colloids Surf. B Biointerfaces.

[75] Soni V.K., Shukla D., Kumar A., Vishvakarma N.K. (2020). Curcumin circumvent lactate-induced chemoresistance in hepatic cancer cells through modulation of hydroxycarboxylic acid receptor-1. Int. J. Biochem. Cell Biol..

[76] Fan H., Tian W., Ma X. (2014). Curcumin induces apoptosis of HepG2 cells via inhibiting fatty acid synthase. Target. Oncol..

[77] Younesian O., Kazerouni F., Dehghan-Nayeri N., Omrani D., Rahimipour A., Shanaki M., Kalkhoran M.R., Cheshmi F. (2017). Effect of Curcumin on Fatty Acid Synthase Expression and Enzyme Activity in Breast Cancer Cell Line SKBR3. Int. J. Cancer Manag..

[78] Bianchi G., Ravera S., Traverso C., Amaro A., Piaggio F., Emionite L., Bachetti T., Pfeffer U., Raffaghello L. (2018). Curcumin induces a fatal energetic impairment in tumor cells in vitro and in vivo by inhibiting ATP-synthase activity. Carcinogenesis.

[79] Sordillo P.P., Helson L. (2015). Curcumin and cancer stem cells: Curcumin has asymmetrical effects on cancer and normal stem cells. Anticancer Res..

[80] Huang Y.-T., Lin Y.-W., Chiu H.-M., Chiang B.-H. (2016). Curcumin Induces Apoptosis of Colorectal Cancer Stem Cells by Coupling with CD44 Marker. J. Agric. Food Chem..

[81] Nakagawa K., Zingg J.-M., Kim S.H., Thomas M.J., Dolnikowski G.G., Azzi A., Miyazawa T., Meydani M. (2014). Differential cellular uptake and metabolism of curcuminoids in monocytes/macrophages: Regulatory effects on lipid accumulation. Br. J. Nutr..

[82] Zingg J.-M., Hasan S.T., Cowan D., Ricciarelli R., Azzi A., Meydani M. (2012). Regulatory effects of curcumin on lipid accumulation in monocytes/macrophages. J. Cell. Biochem..

[83] Han Y., Jo H., Cho J.H., Dhanasekaran D.N., Song Y.S. (2019). Resveratrol as a Tumor-Suppressive Nutraceutical Modulating Tumor Microenvironment and Malignant Behaviors of Cancer. Int. J. Mol. Sci..

[84] Jiang Z., Chen K., Cheng L., Yan B., Qian W., Cao J., Liang C., Wu E., Ma Q., Yang W. (2017). Resveratrol and cancer treatment: Updates. Ann. N. Y. Acad. Sci..

[85] Brockmueller A., Sameri S., Liskova A., Zhai K., Varghese E., Samuel S.M., Büsselberg D., Kubatka P., Shakibaei M. (2021). Resveratrol’s Anti-Cancer Effects through the Modulation of Tumor Glucose Metabolism. Cancers.

[86] Jia L., Gao Y., Zhou T., Zhao X.-L., Hu H.-Y., Chen D.-W., Qiao M.-X. (2021). Enhanced response to PD-L1 silencing by modulation of TME via balancing glucose metabolism and robust co-delivery of siRNA/Resveratrol with dual-responsive polyplexes. Biomaterials.

[87] Shen Y.-A., Lin C.-H., Chi W.-H., Wang C.-Y., Hsieh Y.-T., Wei Y.-H., Chen Y.-J. (2013). Resveratrol Impedes the Stemness, Epithelial-Mesenchymal Transition, and Metabolic Reprogramming of Cancer Stem Cells in Nasopharyngeal Carcinoma through p53 Activation. Evid.-Based Complement. Altern. Med..

[88] Craveiro M., Cretenet G., Mongellaz C., Matias M.I., Caron O., De Lima M.C.P., Zimmermann V.S., Solary E., Dardalhon V., Dulić V. (2017). Resveratrol stimulates the metabolic reprogramming of human CD4+T cells to enhance effector function. Sci. Signal..

[89] Nakaya M., Xiao Y., Zhou X., Chang J.-H., Chang M., Cheng X., Blonska M., Lin X., Sun S.-C. (2014). Inflammatory T Cell Responses Rely on Amino Acid Transporter ASCT2 Facilitation of Glutamine Uptake and mTORC1 Kinase Activation. Immunity.

[90] Cheng Z., Zhang Z., Han Y., Wang J., Wang Y., Chen X., Shao Y., Cheng Y., Zhou W., Lu X. (2020). A review on anti-cancer effect of green tea catechins. J. Funct. Foods.

[91] Wei R., Mao L., Xu P., Zheng X., Hackman R.M., Mackenzie G.G., Wang Y. (2018). Suppressing glucose metabolism with epigallocatechin-3-gallate (EGCG) reduces breast cancer cell growth in preclinical models. Food Funct..

[92] Zhang Z., Zhang S., Yang J., Yi P., Xu P., Yi M., Peng W. (2020). Integrated transcriptomic and metabolomic analyses to characterize the anti-cancer effects of (−)-epigallocatechin-3-gallate in human colon cancer cells. Toxicol. Appl. Pharmacol..

[93] Chu K.O., Chan K.P., Chan S.O., Ng T.K., Jhanji V., Wang C.-C., Pang C.P. (2018). Metabolomics of Green-Tea Catechins on Vascular-Endothelial-Growth-Factor-Stimulated Human-Endothelial-Cell Survival. J. Agric. Food Chem..

[94] Wu K.-H., Ho C.-T., Chen Z.-F., Chen L.-C., Whang-Peng J., Lin T.-N., Ho Y.-S. (2018). The apple polyphenol phloretin inhibits breast cancer cell migration and proliferation via inhibition of signals by type 2 glucose transporter. J. Food Drug Anal..

[95] Lin S.-T., Tu S.-H., Yang P.-S., Hsu S.-P., Lee W.-H., Ho C.-T., Wu C.-H., Lai Y.-H., Chen M.-Y., Chen L.-C. (2016). Apple Polyphenol Phloretin Inhibits Colorectal Cancer Cell Growth via Inhibition of the Type 2 Glucose Transporter and Activation of p53-Mediated Signaling. J. Agric. Food Chem..

[96] Patel B.B., Ackerstaff E., Serganova I.S., Kerrigan J.E., Blasberg R.G., Koutcher J.A., Banerjee D. (2017). Tumor stroma interaction is mediated by monocarboxylate metabolism. Exp. Cell Res..

[97] Přenosil J.E., Kut Ö.M., Dunn I.J., Heinzle E. (2009). Biocatalysis, 2. Immobilized Biocatalysts. Ullmann’s Encycl. Ind. Chem..

[98] Guo C., He J., Song X., Tan L., Wang M., Jiang P., Li Y., Cao Z., Peng C. (2019). Pharmacological properties and derivatives of shikonin—A review in recent years. Pharmacol. Res..

[99] Boulos J.C., Rahama M., Hegazy M.-E.F., Efferth T. (2019). Shikonin derivatives for cancer prevention and therapy. Cancer Lett..

[100] Zhao X., Zhu Y., Hu J., Jiang L., Li L., Jia S., Zen K. (2018). Shikonin Inhibits Tumor Growth in Mice by Suppressing Pyruvate Kinase M2-mediated Aerobic Glycolysis. Sci. Rep..

[101] Liu B., Jin J., Zhang Z., Zuo L., Jiang M., Xie C. (2019). Shikonin exerts antitumor activity by causing mitochondrial dysfunction in hepatocellular carcinoma through PKM2–AMPK–PGC1α signaling pathway. Biochem. Cell Biol..

[102] Tao T., Su Q., Xu S., Deng J., Zhou S., Zhuang Y., Huang Y., He C., He S., Peng M. (2019). Down-regulation of PKM2 decreases FASN expression in bladder cancer cells through AKT/mTOR/SREBP-1c axis. J. Cell. Physiol..

[103] Wang H., Tang Y., Fang Y., Zhang M., Wang H., He Z., Wang B., Xu Q., Huang Y. (2019). Reprogramming Tumor Immune Microenvironment (TIME) and Metabolism via Biomimetic Targeting Codelivery of Shikonin/JQ1. Nano Lett..

[104] Arnison P.G., Bibb M.J., Bierbaum G., Bowers A.A., Bugni T.S., Bulaj G., Camarero J.A., Campopiano D.J., Challis G.L., Clardy J. (2013). Ribosomally synthesized and post-translationally modified peptide natural products: Overview and rec-ommendations for a universal nomenlature. Nat. Prod. Rep..

[105] Dahlem C., Siow W.X., Lopatniuk M., Tse W.K.F., Kessler S.M., Kirsch S.H., Hoppstädter J., Vollmar A.M., Müller R., Luzhetskyy A. (2020). Thioholgamide A, a New Anti-Proliferative Anti-Tumor Agent, Modulates Macrophage Polarization and Metabolism. Cancers.

[106] Liu M., Luo F., Ding C., Albeituni S., Hu X., Ma Y., Cai Y., McNally L.R., Sanders M.A., Jain D. (2015). Dectin-1 Activation by a Natural Product β-Glucan Converts Immunosuppressive Macrophages into an M1-like Phenotype. J. Immunol..

[107] Carrasco-Pozo C., Ni Tan K., Avery V.M. (2020). Hemin Prevents Increased Glycolysis in Macrophages upon Activation: Protection by Microbiota-Derived Metabolites of Polyphenols. Antioxidants.

[108] Ma J.-L., Yang P.-Y., Rui Y.-C., Lu L., Kang H., Zhang J. (2007). Hemin modulates cytokine expressions in macrophage-derived foam cells via heme oxygenase-1 induction. J. Pharmacol. Sci..

[109] Tang W., Song H., Cai W., Shen X. (2015). Real Time Monitoring of Inhibition of Adipogenesis and Angiogenesis by (−)-Epigallocatechin-3-Gallate in 3T3-L1 Adipocytes and Human Umbilical Vein Endothelial Cells. Nutrients.

[110] Su T., Huang C., Yang C., Jiang T., Su J., Chen M., Fatima S., Gong R., Hu X., Bian Z. (2020). Apigenin inhibits STAT3/CD36 signaling axis and reduces visceral obesity. Pharmacol. Res..

[111] Pérez-Jiménez A., Rufino-Palomares E.E., Fernández-Gallego N., Ortuño-Costela M.C., Reyes-Zurita F.J., Peragón J., Salguero E.L.G., Mokhtari K., Medina P.P., Lupiáñez J.A. (2016). Target molecules in 3T3-L1 adipocytes differentiation are regulated by maslinic acid, a natural triterpene from Olea europaea. Phytomedicine.

[112] Nieman K.M., Romero I.L., Van Houten B., Lengyel E. (2013). Adipose tissue and adipocytes support tumorigenesis and metastasis. Biochim. Biophys. Acta Mol. Cell Biol. Lipids.

[113] Rodrigues J., Heinrich M.A., Teixeira L.M., Prakash J. (2021). 3D In Vitro Model (R)evolution: Unveiling Tumor–Stroma Interactions. Trends Cancer.

[114] Khan T., Gurav P. (2018). PhytoNanotechnology: Enhancing Delivery of Plant Based Anti-cancer Drugs. Front. Pharmacol..

